# Analyses of Crime Patterns in NIBRS Data Based on a Novel Graph Theory Clustering Method: Virginia as a Case Study

**DOI:** 10.1155/2014/492461

**Published:** 2014-03-20

**Authors:** Peixin Zhao, Marjorie Darrah, Jim Nolan, Cun-Quan Zhang

**Affiliations:** ^1^School of Management, Shandong University, Jinan, Shandong, China; ^2^Department of Mathematics, West Virginia University, Morgantown, WV, USA; ^3^Department of Sociology and Anthropology, West Virginia University, Morgantown, WV, USA

## Abstract

This paper suggests a novel clustering method for analyzing the National Incident-Based Reporting System (NIBRS) data, which include the determination of correlation of different crime types, the development of a likelihood index for crimes to occur in a jurisdiction, and the clustering of jurisdictions based on crime type. The method was tested by using the 2005 assault data from 121 jurisdictions in Virginia as a test case. The analyses of these data show that some different crime types are correlated and some different crime parameters are correlated with different crime types. The analyses also show that certain jurisdictions within Virginia share certain crime patterns. This information assists with constructing a pattern for a specific crime type and can be used to determine whether a jurisdiction may be more likely to see this type of crime occur in their area.

## 1. Introduction

The National Incident-Based Reporting System (NIBRS) is a crime reporting program for local, state, and federal law enforcement agencies that provides a wealth of incident level data for use in analysis. It is part of the Uniform Crime Reporting (UCR) Program which is administered by the FBI. The UCR Program provides a nationwide view of crime based on data submitted through state programs or directly to the national UCR Program and has been operational for around 70 years. The NIBRS was implemented in the late 1970s to meet law enforcement need for the 21st century. This vast system houses information on offenses, victims, offenders, property, and persons arrested, as well as the incident itself. The data of NIBRS are well structured and readily available for researchers and law enforcement agencies to assist with understanding the intricate nature of crime.

Akiyama and Nolan [1] outlined the structure of the NIBRS data set and provided methods for understanding and analyzing the data. Dunn and Zelenock [2] also describe and test procedures to facilitate the use of this vast system. Building on these initial works, many authors continue to investigate how this storehouse of data can be turned into useful information for researchers and law enforcement agencies. Much of the work employs descriptive statistics applied in various sophisticated ways to extract information from the files. For example, Thompson et al. [3] apply descriptive statistics to examine intimate partner violence and make connections between this crime and other crimes that occurred in the same incident. They were able to show a link between intimidation and more serious violent crimes and extract information about the relationships between the victim and the offender from the NIRBS data that helps to better understand this type of crime. Snyder [4] used logistic regression techniques to predict the arrest of juvenile robbery offenders. More recently, Addington and Rennison [5] used logistic regression models along with the NIBRS and the National Crime Victimization Survey (NCVS) data for predicting rape cooccurrence to provide a critical initial look at rapes that occur with other crimes.

For criminologists the NIBRS data holds the answers to many long-standing questions about crime, criminal offending, and crime victimization. However, gaining access to some of these answers has remained difficult because of the size and complexity of the data. Effective techniques, such as data mining and clustering, for criminal justice data are of increasing importance to both the research and law enforcement communities [6]. In recent years, clustering categorical data has gained more importance because it is one of the fundamental methods in data mining [7]. Clustering crime data, as with other categorical data, is unsupervised learning that aims at partitioning a data set into groups of similar items. The goal is to create clusters of data objects where the within-cluster similarity is maximized and the between-cluster similarity is minimized. Over the years, many clustering algorithms have been developed and tested. Some clustering techniques have developed specifically for use with categorical data. Abdu [8] presented three new clustering algorithms that were applied to the clustering of the NIBRS data. Two of his approaches combine spectral analysis and clustering techniques that are scalable to large data sets such as the NIBRS.

Clustering categorical data poses a challenge not encountered in clustering numerical data because the attribute categories are not ordered and defining a metric with which to measure the distance between data objects in a data set becomes a challenge. Many of the algorithms that have emerged for clustering categorical data rely on the occurrence/cooccurrence frequencies of attribute values in the data set to determine clusters of similar data objects. The basic goal is to choose a set of attribute categories that provide a summary of the data objects in a cluster. There are a wide range of clustering algorithms for categorical data, including K-modes [9], STIRR [10], CACTUS [11], ROCK [12], COOLCAT [13], LIMBO [14], and CLICKS [15]. A good summary can be found in [8]. The clustering algorithm used in this research is a mathematically well-defined model implemented in a polynomial time algorithm that guarantees an optimal solution [16].

Due to the lack of well-defined mathematical models and optimization goals, most existing graph theory clustering approaches could not guarantee a proper clustering result in general cases. For example, agglomerative hierarchical clustering methods could not produce proper clusters with larger sizes, while divisive hierarchical methods could not produce clusters with smaller sizes, and clusters with large difference in their sizes and k-core method may produce clusters with small edge-cuts, and so forth. Many papers and articles have mentioned these problems and frustration among users (e.g., see [17–19]). Even the most popular commercial software, SAS, is unable to produce proper outputs for some simple data.

The purpose of this paper is to present a novel multidimensional clustering method for the NIBRS data. We firstly outlines a new measure, called the* likelihood index*, that helps examine quantitatively how likely a crime is to occur in a particular jurisdiction. This measure compares a vector that describes a jurisdiction with a vector that represents a crime type. Then according to the defined distance between these two vectors, we can determine how closely the jurisdiction aligns with that crime type. The data used in this study were obtained from the 2005 NIBRS which is stored at the National Archive for Criminal Justice Data at the University of Michigan. This work explores the following research questions. Do specific crimes exhibit certain quantifiable characteristics? Do different types of crimes share similar quantifiable characteristics? Do jurisdictions of a state cluster with respect to different crime types? What is the likelihood that if one type of crime is occurring in an area, other types of crime with similar quantifiable characteristic will also occur in that area?

The rest of the paper is organized as follows. In Section 2 we summarize the data unit of analysis and preparation. In Section 3 we introduce the methods to deal with the data matrix and take Virginia as a case study.  Section 4 provides some additional results and Section 5 gives the conclusions of the research.

## 2. Data Unit of Analysis and Preparation

The data sets available in the NIBRS provide a wealth of incident level data about each reported crime. As for 2010, approximately 40 states contribute their data to the massive data set. The data and tools are made available by University of Michigan for use by law enforcement agencies and researchers. In order to devise a manageable set of data for preliminary testing of techniques and for preliminary data analysis, only the 2005 data on assaults were explored. From the 2005 assault data, 121 jurisdictions (counties or cities) in Virginia were selected for examination. These represent all jurisdictions within Virginia with populations greater than 10,000. There were 10,183 incidents reported in these 121 chosen jurisdictions.

For this study, 21 indexes from the NIBRS were chosen from the 246 available indexes. These 21 indexes were deemed important to provide the relevant characteristics of the victim(s), offender(s), and the circumstances of each incident. The selected particular indexes were listed in Table 1.

In order to facilitate the selected analysis techniques, the data was expanded from one column, with many possible entries, to multi columns that contained zero or one. For example, the Offender Segment index contains the sex of the offender and has the possible entries of male, female, or unknown. This index column was split into three individual columns where an entry in the three columns of (1 0 0) means female, (0 1 0) means male, and (0 0 1) means unknown. This turns the column for sex of the offender to three columns. All created columns were binary (0/1) columns that were used to help classify the characteristics of the incident. From the expansion of the original 21 indexes, 57 binary columns were created. This led to the creation of a 121 × 57 Crime Data Matrix, where each row *i* represents the *i*th jurisdiction and each column *j* represents the *j*th parameter related to the incident (e.g., offender sex, victim resident status) or a crime type (e.g., hate crime, drug dealing). The Crime Data Matrix construction is pictured below:

(1) $$\begin{array}{c} \text{Crime}\;\text{Data}\;\text{Matrix}=\left[\begin{array}{ccc} a_{1,1} & \ldots & a_{1,57}\\ \vdots & \ddots & \vdots \\ a_{121,1} & \ldots & a_{121,57} \end{array}\right], \end{array}$$

where *a*_*i*,*j*_ is the entry of the *j*th crime index from the *i*th jurisdiction.

Normalization of the rows of the matrix was completed by dividing each row entry by the population of the jurisdiction. This gave a per person rate for each crime parameter or each crime type. Normalization of the columns was completed by averaging the columns and subtracting the average from each entry in the column. Then each entry in the column was divided by the vector length of the column. Equation (2) shows these normalization step-by-step operations.

(2)  ai,j⟵number  of  occurances  of  crime  parameter  or  crime  typepopulation  of  the  jurisdiction,ai,j⟵ai,j−gj where  gj  is  the  average  of  the  jth  column, ai,j⟵  ai,jnj where  nj  is  the  vector  length  of  the  jth  column.

## 3. Finding Patterns in the Data

This section explains several different analyses that were performed on the data in the matrix described above in order to attempt to answer the research questions listed in Section 1. These analyses include comparing the columns of the matrix to determine the correlation of crime parameters to crime types and to determine the correlation of different crime types. Also there were two analyses performed comparing the row vectors to develop the likelihood index and to cluster the jurisdictions by crime types.

### 3.1. Correlation of Crime Parameters and Crime Types (Comparing the Column Vectors)

The motivation for comparing the different crime parameters to crime types is to determine if there are some characteristics that can tell us about the likelihood of a crime type to occur in a certain jurisdiction. Each crime type may have factors that contribute to a specific crime appearing in a certain place. An overall increase in crime in an area may or may not correlate to an increase in any one particular type of crime, say hate crime, in that area. However, there may be individual parameters whose increase may indicate an increase in a particular type of crime. For example, if juvenile offenders are up in a certain area, this may indicate that hate crimes will also be up in that area. Also crime type vectors were compared against each other in a similar way. For example, crimes like juvenile gang were compared against hate crime to see if these crime types also have a correlation.

In order to determine the relationship between the *j*th parameter (*j*th column of the Crime Data Matrix) and any other column (another parameter or another crime type), the correlation coefficient (cosine of the angle between the two column vectors over the norm of the vectors) was calculated. Each of the columns of the crime data matrix forms a vector in a 121-dimensional space and the vectors can be geometrically compared, with the correlation between two parameters represented by the cosine of the angle in this space. For example, the column for the juvenile offender could be compared against the column for hate crime or the column for hate crime can be compared to the column for gang-related crime. These comparisons are made by calculating the angle between these two columns to determine if there is any relationship and how strong that relationship may be. This method can assist in determining whether two columns vary directly, inversely, or separately.

For this comparison of two column vectors, the variation in two vectors must be transformed to eliminate the effects of mean differences. Once the mean deviation is determined, then the correlation can be determined by the cosine of the angle between the vectors. As an example, let *j* = (*j*_1_, *j*_2_,…, *j*_121_) be the column for juvenile offender and let *h* = (*h*_1_, *h*_2_,…, *h*_121_) be the column for the hate crime indicator. To find the correlation coefficient of this two columns calculate the following:

(3) $$\begin{array}{c} \alpha_{j}=\cos\theta_{j}=\;\frac{\sum_{i}j_{i}h_{i}}{||j||||h||}. \end{array}$$

The sign of the answer is ignored, since either a strong positive relationship (close to 1 meaning that the two angles are in the same direction and close to one another) or a strong negative relationship (close to −1 meaning that the two angles are in opposite directions, but nearly opposite one another) indicates that the vectors appear to be related in some way.

In doing this comparison, with one vector fixed and comparing it to all other vectors and itself, we form a row vector of size 57. Consider the example that compares the hate crime vector with all other vectors. We construct another vector that we refer to as the Hate Crime Character Vector of size 57, which contains all the cosine *θ*_*j*_ (the correlation coefficient of the *j*th parameter with respect to hate crime). A new vector is formed from all these correlation coefficients (*α*_1_, *α*_2_,…, *α*_57_), where each of the 57 parameters has a correlation coefficient vector associated with it. Now consider the corresponding components in the Hate Crime Character Vector.  Table 2 tells us how hate crimes are related to aggravated assault crimes. Among those crimes, juvenile gang (0.4058) has higher correlation with hate crime, while the others, for example, argument (0.1325) and drug dealing (0.0265) have relatively lower correlation.

Table 3 also shows similar evidence that for victim's age, the age group less than 18 is also more correlated to hate crime than the other two age groups. Many such comparisons can be observed using these correlation coefficient vectors.

### 3.2. Analyses of Jurisdictions (Comparing the Row Vectors)

By using the correlation coefficients, a 57-dimensional vector, two data analyses can be performed, each of which indicates the likelihood of a particular crime for each jurisdiction. The first one is a numerical index, called the* likelihood index *assigned to each of the 112 jurisdictions. The second one is a clustering analysis of all jurisdictions. Jurisdictions with similar recorded crime patterns (adjusted corresponding to correlation coefficients) form clusters.

Let *β* = {*β*_1_, *β*_2_, …, *β*_121_} be the* likelihood index vector* where *β*_*i*_ is the likelihood index between jurisdiction *i* and a particular crime (e.g., hate crime) or crime parameter (e.g., juvenile offender). For this comparison of two row vectors, the variation in two vectors must be transformed to eliminate the effects of mean differences. Once the mean deviation is determined, then the correlation can be determined by the cosine of the angle between the vectors. As an example, let *k* = (*k*_1_, *k*_2_,…, *k*_57_) be the row for the Norfolk jurisdiction and let *l* = (*l*_1_, *l*_2_,…, *l*_57_) be the row for the hate crime indicator. To find the correlation coefficient of these two rows calculate the following:

(4) $$\begin{array}{c} \beta_{i}=\cos\phi_{i}=\frac{\sum_{j}^{}k_{j}l_{j}}{||k||||l||}. \end{array}$$

Table 4 gives the top 30 jurisdictions of Virginia with respect to the hate crime likelihood index and also provides the data on the clustering of the jurisdictions discussed in the next section.

### 3.3. Using the Correlation Coefficients to Cluster the Jurisdictions

**Figure 1:**
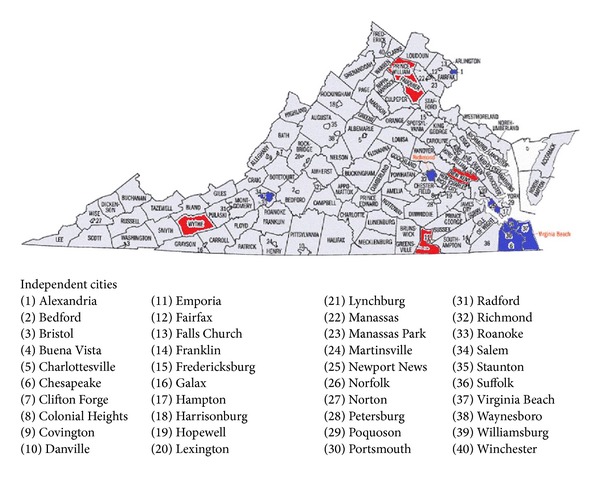
The clustering results of 121 counties in Virginia according to hate crime.

To begin the clustering, a weighted complete graph with 121 vertices is formed. The weight on each edge is the correlation coefficient between the jurisdictions. The novel graph theory clustering method we proposed in [20] is used to find all the dense (highly weighted) subgraphs of the complete graph. A distinguished feature of this method, nonbinary hierarchical tree, clearly highlights meaningful clusters which significantly reduces further manual efforts for cluster selections. The results of clustering the jurisdictions with respect to hate crimes are also displayed in Figure 1 and Table 4.

It can be seen that of the top 30 jurisdictions with respect to hate crimes, 12 of the top 15 are in Cluster A and the others are in Cluster B. The remaining 91 jurisdictions also fall into Cluster B. The 12 higher hate crime rate counties (cities) are showed in Figure 1 (in red and blue).

## 4. Additional Results

Similar analyses where performed with respect to other crime types: drug-dealing, juvenile gang, and gangland (organized crime involvement). The results are summarized in Tables 5–10.

Table 6 summarizes the drug dealing vector comparison with the offender parameter vectors.

Tables 5 and 6 summarize the comparison of the drug dealing vector with other crime parameter vectors. Each of the other crime parameters related to the victim and the offender was compared to the drug dealing vector. A high value for this correlation coefficient implies that there is a correlation between this crime parameter and drug dealing. Again, the motivation for comparing the different crime parameters to crime types is an attempt to determine if there are some characteristics that can tell us about the likelihood of a crime type to occur in a certain jurisdiction. For example, the correlation between the drug dealing vector and individual victim is 0.5789, which is much higher than the correlation between drug dealing and victim type business (0.1721) or victim type society/public (0.1729). This would seem to imply that for jurisdictions where the individual victim crimes are evident they may also have the likelihood for drug dealing related crimes. The tables show the correlation coefficient values: the correlation values in bold show a higher correlation with the drug dealing crime type than the other parameters in that category.

Table 7 summarizes the drug dealing vector comparison with the other crime types. The highest correlation is with argument.

Each of the other crime parameters related to the victim and the offender was compared to the gangland (organized crime involvement) vector. There were no significant relationships to report from this comparison.

Table 8 gives the top 30 jurisdictions of Virginia with respect to the drug dealing likelihood index and also shows how the jurisdictions are clustered. Given the 121 Virginia jurisdictions being considered, of the top 30 with respect to the likelihood index, twelve are clustered together. Only three others jurisdictions from Cluster B appear outside the top 30; these jurisdictions are Smyth (35), Tazewell (37), and Pittsylvania (50). Each of the other crime parameters related to the victim and the offender was compared to the gangland (organized crime involvement) vector. There were no significant relationships to report from this comparison.

Table 9 gives the top 30 jurisdictions of Virginia with respect to the gangland (organized crime involvement) likelihood index and also shows how the jurisdictions are clustered. Given the 121 Virginia jurisdictions being considered, of the top 30 with respect to the likelihood index, thirteen are clustered together. Only one other jurisdiction from cluster B appears outside the top 30; this one jurisdiction is Fairfax County PD (31). Each of the other crime parameters related to the victim and the offender was compared to the juvenile gang vector. There were no significant relationships to report from this comparison.

Table 10 gives the top 30 jurisdictions of Virginia with respect to the juvenile gang likelihood index and also shows how the jurisdictions are clustered. Given the 121 Virginia jurisdictions being considered, of the top 30 with respect to the likelihood index, sixteen are clustered together. No other jurisdiction from Cluster B appears outside the top 30.

## 5. Conclusion

The NIBRS provides a wealth of incident level data for use in analysis. The methods investigated in this research yielded promising preliminary results. The methods were applied only to the assault data from 2005 but can easily be extended to other crime types and to other years to validate these results and also provide longitudinal investigation.

The comparison between the crime type vector and the individual parameters vectors helped in two cases (hate crimes and drug dealing) to determine which factors was more related to those crimes. The different types of analyses that were conducted on the jurisdictions helped to validate one another. The likelihood index looked at whether a certain crime pattern existed in that jurisdiction, while the clustering method sought to cluster all the jurisdictions based on the crime patterns of that jurisdiction. This information could be useful to assist law enforcement agencies or policy makers in determining which jurisdictions share common challenges that could possibly be addressed through cooperation and sharing resources between jurisdictions.

The next steps would be to utilize this same approach for data from other states or perhaps a larger region to examine if the same information is observed from the analyses. It will be interesting to see if Virginia data and other states have the same patterns or if different patterns emerge. Further research and refinement of these methods should yield tools that would provide researchers, law enforcement agencies, and government officials with a means to find patterns of different crime types and possibly identify jurisdictions that may be likely to experience that type of crime.

## Figures and Tables

**Table 1:** NIBRS indexes used.

Segment	Index	Number of subindexes
Victim indexes	Type of victim	3
	Victim age	3
	Victim sex	2
	Victim race	5
	Victim ethnicity	3
	Victim residence status	3
	Aggravated assault/homicide circumstances	10
	Type of injury	6

Offender indexes	Offender age	3
	Offender sex	3
	Offender race	5

Additional indexes	Injury	2
	Juvenile	1
	Violent crime	1
	Juvict	1
	Multiple victims	1
	Multiple offenders	1
	Multiple offenders and victims	1
	Multiple offenders and one victim	1
	One offender and multiple victims	1
	One offender and one victims	1

**Table 2:** Hate crime correlation coefficient vector values for other crimes.

Crime type	Hate crime correlation coefficient (*α*_*j*_)
Argument	0.1325
Assault on law enforcement officers	0.0371
Drug dealing	0.0265
Gangland (organized crime involvement)	0.1565
Juvenile gang	**0.4058**
Lovers' quarrel	−0.0281
Other felony involved	−0.0375
Other circumstances	0.0961
Unknown circumstance	0.1356

**Table 3:** Hate crime correlation coefficients for age of victim.

Age of victim	Hate crime correlation coefficient (*α*_*j*_)
Age < 18	**0.4944**
18 ≤ Age ≤ 60	0.0540
Age > 60	0.0474

**Table 4:** Likelihood index and clustering for hate crime.

	Name	Cluster	Hate crime likelihood index
1	NEWPORT NEWS	**B**	0.867
2	NORFOLK	**B**	0.837
3	CHESAPEAKE	**B**	0.810
4	GREENSVILLE	**B**	0.762
5	PORTSMOUTH	**B**	0.759
6	RICHMOND	**B**	0.716
7	WYTHE	**B**	0.681
8	ALEXANDRIA	**B**	0.678
9	BRISTOL	**B**	0.667
10	NEW KENT	**B**	0.618
11	FAUQUIER	**B**	0.531
12	ROANOKE	A	0.439
13	RICHMOND	A	0.386
14	WILLIAMSBURG	A	0.377
15	VIRGINIA BEACH	**B**	0.318
16	CHARLOTTESVILLE	A	0.312
17	PETERSBURG	A	0.280
18	SPOTSYLVANIA	A	0.226
19	HOPEWELL	A	0.205
20	RUSSELL	A	0.121
21	CLARKE	A	0.121
22	WINCHESTER	A	0.118
23	SUFFOLK	A	0.111
24	MARTINSVILLE	A	0.075
25	STAUNTON	A	0.073
26	GALAX	A	0.070
27	SHENANDOAH	A	0.063
28	CAROLINE	A	0.044
29	SURRY	A	−0.020
30	DANVILLE	A	−0.094

**Table 5:** Drug dealing correlation with other crime parameters related to victim.

	Drug dealing correlation coefficient
Type of victim	
individual	**0.5789**
Business	0.1721
Society/public	0.1729
Age of victim	
Age < 18	0.2933
Age ≥ 60	0.3872
18 ≤ age < 60	**0.5993**
Sex of victim	
Male	**0.5774**
Female	0.4989
Race of victim	
White	0.3906
Black	**0.5028**
Asia/Pacific Islander	0.0764
Unknown	0.0003
American Indian	0.0393
Ethnicity of victim	
Hispanic origin	0.0688
Not of Hispanic origin	**0.6009**
Unknown	−0.0616
Resident status of victim	
Nonresident	0.2631
Resident	**0.5740**
Unknown	0.1290

**Table 6:** Drug dealing correlation with other crime parameters related to offender.

Offender age	
Age < 18	0.3514
Age ≥ 60	0.3762
18 ≤ age < 60	**0.5678**
Offender sex	
Male	0.5331
Female	**0.5685**
Unknown	0.1972
Offender race	
White	0.3145
Black	**0.5133**
American Indian/Alaskan native	−0.0479
Unknown	0.1948
Asian/Pacific Islander	0.113

**Table 7:** Drug dealing correlation with other crime types.

Other crime types	
Argument	**0.4854**
Assault on law enforcement officer(s)	0.3117
Gangland (organized crime involvement)	0.3456
Juvenile gang	0.0706
Lovers' quarrel	0.2967
Other felony involved	0.2955
Hate crime	0.0265

**Table 8:** Likelihood index and clustering for drug dealing.

	Name	Cluster	Drug dealing likelihood index
1	CHARLOTTESVILLE	**B**	0.9094
2	NEWPORT NEWS	**B**	0.9012
3	CHESAPEAKE	**B**	0.8851
4	PETERSBURG	**B**	0.8637
5	RICHMOND	**B**	0.8365
6	PORTSMOUTH	A	0.8281
7	HOPEWELL	A	0.7917
8	ROANOKE	**B**	0.791
9	SUFFOLK	**B**	0.7839
10	NORFOLK	**B**	0.7756
11	BRISTOL	**B**	0.7365
12	DANVILLE	A	0.707
13	GREENSVILLE	A	0.7032
14	GALAX	A	0.6881
15	CAROLINE	A	0.6852
16	SUSSEX	A	0.6396
17	WINCHESTER	A	0.6327
18	FREDERICKSBURG	**B**	0.6031
19	CLARKE	A	0.6021
20	FRANKLIN	A	0.5808
21	RICHMOND	A	0.5667
22	LYNCHBURG	A	0.5395
23	MECKLENBURG	A	0.5185
24	RADFORD	A	0.5133
25	GOOCHLAND	A	0.5114
26	MANASSAS	A	0.494
27	HENRY	**B**	0.4844
28	NORTON	A	0.4456
29	WISE	**B**	0.4381
30	WILLIAMSBURG	A	0.395

**Table 9:** Likelihood index and clustering for gangland (organized crime involvement).

	Name	Cluster	Gangland likelihood index
1	NEWPORT NEWS	**B**	0.9217
2	ROANOKE	**B**	0.8786
3	CHESAPEAKE	**B**	0.8713
4	PETERSBURG	**B**	0.8421
5	NORFOLK	**B**	0.8129
6	RICHMOND	**B**	0.7953
7	LYNCHBURG	**B**	0.6965
8	FREDERICKSBURG	**B**	0.689
9	BRISTOL	**B**	0.6787
10	ALEXANDRIA	**B**	0.6569
11	NORTHAMPTON	**B**	0.632
12	LOUDOUN	**B**	0.4939
13	CHARLOTTESVILLE	A	0.4152
14	STAFFORD	**B**	0.3853
15	PORTSMOUTH	A	0.3075
16	HOPEWELL	A	0.2724
17	GALAX	A	0.2121
18	CAROLINE	A	0.1372
19	SUFFOLK	A	0.1077
20	GREENSVILLE	A	0.0817
21	CLARKE	A	0.0689
22	DANVILLE	A	0.0519
23	HENRY	A	0.0518
24	RICHMOND	A	0.0074
25	WINCHESTER	A	−0.0071
26	FRANKLIN	A	−0.0508
27	SUSSEX	A	−0.0763
28	MANASSAS	A	−0.0812
29	WILLIAMSBURG	A	−0.1001
30	GOOCHLAND	A	−0.1112

**Table 10:** Likelihood index and clustering for juvenile gang.

	Name	Cluster	Juvenile gang likelihood index
1	NORFOLK	**B**	0.8424
2	ROANOKE	**B**	0.8387
3	RICHMOND	**B**	0.7987
4	PORTSMOUTH	**B**	0.7977
5	GREENSVILLE	**B**	0.7575
6	CHESAPEAKE	**B**	0.7211
7	NEWPORT NEWS	**B**	0.7112
8	WILLIAMSBURG	**B**	0.6639
9	WYTHE	**B**	0.5719
10	ALEXANDRIA	**B**	0.5597
11	LYNCHBURG	**B**	0.5452
12	MARTINSVILLE	**B**	0.5355
13	PULASKI	**B**	0.4884
14	HAMPTON	**B**	0.4848
15	SPOTSYLVANIA	**B**	0.451
16	POWHATAN	**B**	0.442
17	PETERSBURG	A	0.4207
18	CHARLOTTESVILLE	A	0.4064
19	HOPEWELL	A	0.3519
20	RICHMOND	A	0.208
21	GALAX	A	0.2025
22	BRISTOL	A	0.2012
23	SUFFOLK	A	0.1925
24	CAROLINE	A	0.121
25	CLARKE	A	0.0939
26	WINCHESTER	A	0.0893
27	DANVILLE	A	0.0834
28	SUSSEX	A	0.051
29	CAMPBELL	A	0.0119
30	FRANKLIN	A	−0.0773
